# Supplementary material for: Modeling glioblastoma heterogeneity as a dynamic network of cell states
Source: Mol Syst Biol. 2021 Sep 16;17(9):e10105. doi: 10.15252/msb.202010105 (PMC8444284; doi:10.15252/msb.202010105)
Supplement: Supplementary file 6 — Source Data for Figure 5 [file MSB-17-e10105-s004.zip › Figure5A_sourcedata/GSEA_3071/nosigdb_stateIV.GseaPreranked.1622795164626/NB_KI_CLUSTER5.html]

Details for gene set NB\_KI\_CLUSTER5[GSEA]

|  || Dataset | state53071 |
| Phenotype | NoPhenotypeAvailable |
| Upregulated in class | na\_neg |
| GeneSet | NB\_KI\_CLUSTER5 |
| Enrichment Score (ES) | -0.6593644 |
| Normalized Enrichment Score (NES) | -3.260938 |
| Nominal p-value | 0.0 |
| FDR q-value | 0.0 |
| FWER p-Value | 0.0 |
Table: GSEA Results Summary

  

Fig 1: Enrichment plot: NB\_KI\_CLUSTER5      
 Profile of the Running ES Score & Positions of GeneSet Members on the Rank Ordered List

  

| PROBE | GENE SYMBOL | GENE\_TITLE | RANK IN GENE LIST | RANK METRIC SCORE | RUNNING ES | CORE ENRICHMENT || 1 | ARL6IP1 |  |  | 458 | 0.849 | -0.0735 | No |
| 2 | CDCA7 |  |  | 818 | 0.596 | -0.1323 | No |
| 3 | LBR |  |  | 2271 | 0.260 | -0.4063 | No |
| 4 | PTTG1 |  |  | 2470 | -0.266 | -0.4398 | No |
| 5 | CDKN2D |  |  | 2615 | -0.284 | -0.4626 | No |
| 6 | CDC25B |  |  | 2671 | -0.292 | -0.4683 | No |
| 7 | HMGB1 |  |  | 2819 | -0.308 | -0.4913 | No |
| 8 | EZH2 |  |  | 2946 | -0.324 | -0.5100 | No |
| 9 | SMC4 |  |  | 3035 | -0.337 | -0.5212 | No |
| 10 | MXD3 |  |  | 3316 | -0.377 | -0.5685 | No |
| 11 | TUBA1B |  |  | 3357 | -0.383 | -0.5697 | No |
| 12 | DDX11 |  |  | 3527 | -0.415 | -0.5951 | No |
| 13 | CKAP2 |  |  | 3633 | -0.433 | -0.6080 | No |
| 14 | ECT2 |  |  | 3902 | -0.487 | -0.6512 | Yes |
| 15 | MIS18BP1 |  |  | 3913 | -0.490 | -0.6448 | Yes |
| 16 | CIP2A |  |  | 3968 | -0.502 | -0.6467 | Yes |
| 17 | HIST1H1E |  |  | 4011 | -0.515 | -0.6461 | Yes |
| 18 | NCAPD2 |  |  | 4055 | -0.526 | -0.6455 | Yes |
| 19 | TEX30 |  |  | 4076 | -0.532 | -0.6404 | Yes |
| 20 | ZWILCH |  |  | 4082 | -0.534 | -0.6323 | Yes |
| 21 | PLK1 |  |  | 4205 | -0.569 | -0.6462 | Yes |
| 22 | ANP32B |  |  | 4235 | -0.576 | -0.6420 | Yes |
| 23 | HMGB2 |  |  | 4243 | -0.578 | -0.6337 | Yes |
| 24 | BUB3 |  |  | 4249 | -0.580 | -0.6248 | Yes |
| 25 | KPNA2 |  |  | 4361 | -0.621 | -0.6357 | Yes |
| 26 | LIG1 |  |  | 4368 | -0.624 | -0.6263 | Yes |
| 27 | DNMT1 |  |  | 4388 | -0.632 | -0.6193 | Yes |
| 28 | FANCA |  |  | 4403 | -0.635 | -0.6113 | Yes |
| 29 | RHEB |  |  | 4416 | -0.641 | -0.6028 | Yes |
| 30 | SHCBP1 |  |  | 4473 | -0.667 | -0.6023 | Yes |
| 31 | DHFR |  |  | 4517 | -0.688 | -0.5990 | Yes |
| 32 | DBF4 |  |  | 4570 | -0.717 | -0.5969 | Yes |
| 33 | TUBB4B |  |  | 4575 | -0.722 | -0.5855 | Yes |
| 34 | CEP152 |  |  | 4599 | -0.733 | -0.5776 | Yes |
| 35 | CKS2 |  |  | 4600 | -0.733 | -0.5652 | Yes |
| 36 | SPDL1 |  |  | 4617 | -0.741 | -0.5558 | Yes |
| 37 | BIRC5 |  |  | 4618 | -0.745 | -0.5433 | Yes |
| 38 | RACGAP1 |  |  | 4623 | -0.748 | -0.5315 | Yes |
| 39 | DUT |  |  | 4642 | -0.762 | -0.5221 | Yes |
| 40 | SGO2 |  |  | 4650 | -0.764 | -0.5106 | Yes |
| 41 | MCM7 |  |  | 4704 | -0.800 | -0.5073 | Yes |
| 42 | TCF19 |  |  | 4719 | -0.807 | -0.4964 | Yes |
| 43 | TRIP13 |  |  | 4721 | -0.808 | -0.4830 | Yes |
| 44 | RANBP1 |  |  | 4741 | -0.823 | -0.4728 | Yes |
| 45 | RAN |  |  | 4743 | -0.824 | -0.4591 | Yes |
| 46 | KIF20B |  |  | 4745 | -0.825 | -0.4454 | Yes |
| 47 | CHAF1A |  |  | 4751 | -0.828 | -0.4324 | Yes |
| 48 | CHEK1 |  |  | 4764 | -0.837 | -0.4206 | Yes |
| 49 | MCM5 |  |  | 4792 | -0.853 | -0.4115 | Yes |
| 50 | HIST2H2AC |  |  | 4801 | -0.866 | -0.3984 | Yes |
| 51 | CDT1 |  |  | 4813 | -0.880 | -0.3857 | Yes |
| 52 | HELLS |  |  | 4819 | -0.882 | -0.3718 | Yes |
| 53 | MCM3 |  |  | 4834 | -0.901 | -0.3593 | Yes |
| 54 | PRC1 |  |  | 4846 | -0.912 | -0.3461 | Yes |
| 55 | SMC2 |  |  | 4847 | -0.912 | -0.3307 | Yes |
| 56 | PRR11 |  |  | 4868 | -0.934 | -0.3189 | Yes |
| 57 | GMNN |  |  | 4873 | -0.939 | -0.3038 | Yes |
| 58 | HIST1H1C |  |  | 4877 | -0.940 | -0.2886 | Yes |
| 59 | CENPM |  |  | 4881 | -0.947 | -0.2732 | Yes |
| 60 | GINS2 |  |  | 4897 | -0.962 | -0.2599 | Yes |
| 61 | FBXO5 |  |  | 4910 | -0.978 | -0.2457 | Yes |
| 62 | H2AFZ |  |  | 4919 | -0.983 | -0.2307 | Yes |
| 63 | BRCA1 |  |  | 4938 | -1.002 | -0.2173 | Yes |
| 64 | CENPW |  |  | 4978 | -1.056 | -0.2070 | Yes |
| 65 | ATAD2 |  |  | 4980 | -1.058 | -0.1894 | Yes |
| 66 | UBE2T |  |  | 5000 | -1.091 | -0.1747 | Yes |
| 67 | PCNA |  |  | 5060 | -1.214 | -0.1656 | Yes |
| 68 | CENPK |  |  | 5091 | -1.267 | -0.1500 | Yes |
| 69 | ZWINT |  |  | 5115 | -1.321 | -0.1322 | Yes |
| 70 | CKS1B |  |  | 5121 | -1.338 | -0.1106 | Yes |
| 71 | DIAPH3 |  |  | 5161 | -1.488 | -0.0931 | Yes |
| 72 | CDK1 |  |  | 5164 | -1.512 | -0.0680 | Yes |
| 73 | CLSPN |  |  | 5202 | -1.663 | -0.0471 | Yes |
| 74 | PCLAF |  |  | 5209 | -1.698 | -0.0197 | Yes |
| 75 | HIST1H4C |  |  | 5249 | -2.081 | 0.0079 | Yes |
Table: GSEA details [plain text format]

  

Fig 2: NB\_KI\_CLUSTER5: Random ES distribution      
 Gene set null distribution of ES for **NB\_KI\_CLUSTER5**
